# Study on prevalence, clinical presentation, and associated bacterial pathogens of goat mastitis in Bauchi, Plateau, and Edo states, Nigeria

**DOI:** 10.14202/vetworld.2019.638-645

**Published:** 2019-05-07

**Authors:** Faruq Ahmad Danmallam, Nikolai Vasilyevich Pimenov

**Affiliations:** Department of Biology and Pathology of Small Domestic, Laboratory and Exotic Animals, Faculty of Veterinary Medicine, Moscow State Academy of Veterinary Medicine and Biotechnology – MVA by K. I. Skryabin, Moscow, Russia

**Keywords:** bacteria, goats, mastitis, Nigeria, physical feature, prevalence

## Abstract

**Aim:**

This study aimed to estimate the prevalence and clinical presentations of different forms of mastitis and mastitis-causing pathogens in lactating goats in Bauchi, Plateau, and Edo states, Nigeria.

**Materials and Methods:**

A total of 500 quarters from 250 lactating goats of Red Sokoto and West African Dwarf breeds during the lactation period were clinically examined. Clinical mastitis was detected by gross signs of udder infection during physical examination and abnormal milk, whereas subclinical mastitis (SCM) was recognized using California mastitis test. The bacterial pathogens were identified by morphology, hemolysis, gram staining, and biochemical tests such as catalase, oxidase, coagulase, reaction on sulfite, indole, and motile medium, and fermentation of sugars.

**Results:**

The overall prevalence of mastitis in goats was found to be 101 (40.4%), of which 8% (20/250) were clinical, and 32.4% (81/250) were SCM cases. The quarter level prevalence was 29.4% (145/493), comprising 5.9% (29/493) clinical and 23.2% (116/493) subclinical forms of mastitis. In addition, 1.4% (7/500) of teats were found to be blind on the clinical examination of the udder and teat. Several regional inflammatory reactions and abnormalities in milk were found in 69% and 100% of the cases, respectively. Moreover, some indications of generalized signs such as fever, reduction in appetite, increase in respiration, and pulse rate per minute were recorded in 100%, 75%, 85% and 80% of the cases, respectively. The predominant bacterial isolates recovered were *Staphylococcus aureus* (20.0%), followed by *Escherichia coli* (15.5%) and *Streptococcus agalactiae* (11.0%), and the least isolated microorganisms (≤6%) were bacteria of different species including *Staphylococcus auricularis*, *Staphylococcus caprae*, *Staphylococcus chromogenes*, *Staphylococcus epidermidis*, *Staphylococcus hyicus*, *Staphylococcus xylosus*, *Staphylococcus lentus*, *Streptococcus dysgalactiae*, *Streptococcus pluranimalium*, *Streptococcus uberis*, *Streptococcus pneumoniae*, *Streptococcus ruminatorum*, *Streptococcus suis*, *Micrococcus luteus*, *Enterobacter cloacae*, *Proteus vulgaris*, *Klebsiella oxytoca*, *Klebsiella pneumoniae*, *Morganella morganii*, *Salmonella* Typhimurium, *Citrobacter freundii*, *Pseudomonas aeruginosa*, *Acinetobacter rudis*, *Acinetobacter haemolyticus*, and *Bacillus cereus*.

**Conclusion:**

Mastitis continues to be recognized as one of the important health issues and leads to major economic losses to the dairy goats caused by many bacterial pathogens, and the effective measures need to be taken to control the disease.

## Introduction

Small ruminants play a very significant role in the socioeconomic and nutritional requirements of most countries. In Nigeria, many families keep goats as a source of complementary income [1]. Within the past 7 years, the recent FAO statistics [2] puts the number of goats in the world at 861.9 million with 53.8 million goats in Nigeria alone. This represents 6.24% of the total goat population in the world [2]. Goat production and productivity and producers’ benefits are far below expectations due to disease, malnutrition, low genetic potential, and poor management practices. Mastitis and other infectious diseases are the major constraints to goat’s production in Nigeria [3]. Among infectious diseases, mastitis is one of the major diseases affecting dairy goat productivity [4,5].

Mastitis is a parenchymal inflammation of the mammary gland, characterized by physical, chemical, and usually bacteriological changes in milk and pathological changes in glandular tissues [6,7]. In general, mastitis occurs in two forms which include clinical (overt) which was subdivided into serous, catarrhal, purulent-catarrhal, and gangrenous and subclinical (hidden) [8,9]. Clinical mastitis (CM) is characterized by sudden onset of swelling and redness of udder, pain, and reduced and altered milk secretion from the affected quarters. The milk may contain clots or flakes or become watery in consistency accompanied by fever, depression and anorexia [10]. Mastitis is a major cause of substantial economic losses in dairy goat’s flocks. Economic losses are due to decreased milk yield, changes in milk quality and composition, increased cost of treatment, increased mortality of kids, and potentially premature culling of small ruminants [11,12].

The predominant pathogens for mastitis throughout the world include *Staphylococcus*, *Streptococcus*, and coliform species [13]. In Nigeria, several etiological agents of mastitis in small ruminants have been reported, such as *Staphylococcus aureus*, *Staphylococcus epidermidis*, *Streptococcus agalactiae*, *Streptococcus dysgalactiae*, *Klebsiella pneumoniae*, *Escherichia coli*, *Salmonella* Typhimurium, *Shigella flexneri*, *Proteus vulgaris*, *Proteus mirabilis*, *Bacillus* spp., *Corynebacterium pseudotuberculosis*, and *Pseudomonas aeruginosa* [14,15]. Predisposing factors such as poor management and hygiene, teat injuries, and wrong milking methods are known to play an important role in the invading of bacterial pathogen in the glands and occurrence of disease [16].

This study aimed to estimate the prevalence and clinical presentations of different forms of mastitis and mastitis-causing pathogens in lactating goats in Bauchi, Plateau, and Edo states, Nigeria.

## Materials and Methods

### Ethical approval

All the animals in this study were treated according to the ethical standard of Moscow State Academy of Veterinary Medicine and Biotechnology – MBA named after K. I. Skryabin. The experiment was carried out in accordance with the guidelines laid down by the International Animal Ethics Committee and in accordance with Local Laws and Regulations.

### Study area

**Figure-1:**
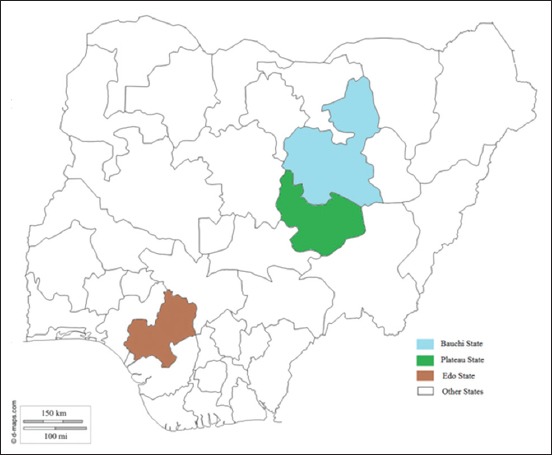
Map of Nigeria showing the location of states of the study areas for the study. (Source: https://pasarelapr.com/detail/blank-map-of-nigeria-9.html).

This study was carried out in 54 herds selected (at random from three Nigerian States) (Figure-1), namely: Bauchi state is one of the six states of the northeastern region of Nigeria. It is located between latitudes 9°3’ and 12°3’ and longitude 8°5’ and 11° east. Bauchi state occupies a total land area of 49,119 km^2^ representing about 5.3% of Nigeria’s total landmass. Bauchi state has an estimated population of 4,653,066 people with 20 local government area councils [17]. The state has a small ruminant population of 2.8 million sheep and 3.4 million goats [18].

Plateau state is located in Nigeria’s Middle Belt. It is located between latitude 08°24’N and longitude 008°32’ and 010°38’ east. Plateau state occupies a total land area of 26,899 km^2^. The state has an estimated population of 3,206,531 people with 17 local government area councils [17]. The state has a small ruminant population of 964,188 sheep and 1,865,805 goats [19].

Edo state is situated in the mid-southwest of Nigeria. It is located between latitude 6.5438° N and longitude 5.8987° E. The state covers 17,802 km^2^. Edo State has estimated population of 3,233,366 people with 18 local government area councils [17].

### Study animals

The animals were lactating goats including Red Sokoto and West African Dwarf breeds, managed under extensive or semi-intensive system.

### Study design and sample size determination

A cross-sectional study was used to screen mastitis in lactating goats. The sample size was determined according to Thrusfield [20] formula with an expected prevalence of 12.65% [15] and 95% statistical confidence level. Accordingly, the sample size of lactating goats was determined to be 170 based on the formula; however, for the purpose of this study, a total of 250 lactating goats, 125 each from extensive and semi-intensive system, were included in the study. In general, 54 herds, of which 36 were small-holders (owning <5 lactating goats) and 18 from medium-sized (owned ≥5 lactating goats) owned by private individual owners, were included in the study. The basis for sampling was the production system (extensive or semi-intensive) practiced by individual farmers in the study area. Extensive system was defined as production system where goats are left to wander and graze during the day and are enclosed during the night, whereas in semi-intensive system, the goats are limited to grazing during the day, fed with dry legume hay (pasture) and supplemented with a local concentrate popularly known as dusa-massara (maize offal), and also enclosed during the night. A general study was carried out, then systemic clinical and special studies (inspection and palpation of the udder, forestriping test and microscopic evaluation of milk), and laboratory test of the milk (culturing, isolation and biochemical identification of the isolated microorganisms).

### Clinical examination

The udders were carefully inspected followed by thorough palpation to detect temperature, possible fibrosis, inflammatory swellings, visible injury, tick infestation, atrophy of the tissue, and swelling of supramammary lymph nodes. Rectal temperature was measured by a digital clinical thermometer (Model ICO Technology “mini color,” Barcelona, Spain; range, 32-43.9°C; accuracy, 0.1°C, pulse rate (PR) was taken from the saphenous artery, whereas the number of inhalations and exhalations during 60 s indicated the respiration rate. During the study, 20 clinically affected goats (with 29 udders: Both halves – 20 and one halve – 9) were studied in detail. The clinical cases of mastitis observed were classified as serous, catarrhal, and purulent-catarrhal [21] and defined as follows.

#### Serous mastitis

Warm, swollen, hyperemic, painful mammary gland with abnormalities of the milk. Affected animals may show signs of depression, fever, and recumbency but are not usually as severely affected as those with peracute mastitis [22]. Supramammary lymph nodes were 1.5-2 times enlarged and painful to palpation.

#### Catarrhal or peracute mastitis

Less apparent inflammation of the mammary gland, but with abnormalities in milk. Affected animals may show signs of depression, fever, and dehydration and frequently become recumbent [23]. Supramammary lymph nodes were enlarged and painful to touch.

#### Purulent-catarrhal

Depression and fever. The affected udders were swollen, hard, painful, and hot, with a grossly enlarged teat. Bluish-reddish tinge and spotty hemorrhages may be visible on the skin. Supramammary lymph nodes were enlarged and painful. The secretion from the affected udder is usually thick with foul-smelling yellow-green pus [21].

### Diagnosis of subclinical mastitis (SCM)

The California mastitis test (CMT) was used to detect the presence of SCM. This screening test was performed according to the procedure given for mastitis by Quinn *et al*. [24]. The result was scored as 0, +1, +2, or +3 depending on the intensity of reaction. Samples with CMT result score of 0 and +1 were considered as negative, while those with a score of +2 or +3 were taken as positive.

### Milk sample collection

Milk samples were collected aseptically from 493 quarters (250 lactating goats) diagnosed with CMT >+1 and were submitted for bacteriological examination. Briefly, the udder was thoroughly washed with water and dried with a clean towel. After disinfecting the teats with 70% ethyl alcohol swabs, milk was collected. The first 3-4 streams of milk were discarded, and then, 5-10 ml of milk was collected from each teat aseptically in separate universal bottles which held a slightly horizontal position to avoid contamination from the udder [25]. Immediately, after collection, samples were cooled and transferred to the laboratory on ice and were stored at −20°C until analyzed.

### Bacteriological examination of milk samples

The isolation and identification of bacterial pathogens were performed according to the procedure described by Mbindyo *et al*. [26]. In Brief, 0.1 ml milk sample was cultured on blood agar, nutrient agar, MacConkey agar, and Brucella agar base. The inoculated plates were incubated at 37°C aerobically for 24-48 h, while those on Brucella agar were incubated at 37°C anaerobically for 7 days. The bacterial pathogens were identified by morphology, hemolysis, gram staining, and biochemical tests such as catalase, oxidase, coagulase, reaction on sulfite, indole, and motile medium, and fermentation of sugars.

### Statistical analysis

Data obtained from CMT and physical examinations were used to determine the apparent prevalence of SCM and CM (blind teat) in goats, respectively. Apparent prevalence of mastitis, clinical presentation, and isolated pathogens was determined using standard formulae, i.e., the number of positive animal/samples divided by the total of animals/samples examined.

## Results

Of the total 250 examined lactating goats, 101 (40.4%; 95% confidence interval [CI]: 34.3-46.8) goats were diagnosed positive for mastitis. Of this, 20 (8%; 95% CI: 5.1-12.3) were clinical mastitis and 81 (32.4%; 95% CI: 26.7-38.6) were subclinical cases. From the functional 493 teats (samples) examined, 145 were diagnosed positive for mastitis, which is 29.4% (95% CI: 25.5-33.7), of which 116 (23.5%; 95% CI: 19.9-27.6) milk samples showed CMT score of either +2 or +3, indicating the presence of SCM and clinical cases in 29 (5.9%; 95% CI: 4.0-8.4) udder halves. Of 500 quarters examined, 7 (1.4%; 95% CI: 0.6-3.0) teats were found blind (Table-1).

**Table-1:** The degree of prevalence of mastitis at individual animal and quarter level in lactating goats in three Nigerian States.

Number of animals examined	Number of quarter/samples examined	Number of goats/quarters with subclinical forms (%)	95% CI	Number of goats/quarters with clinical forms (%)	95% CI	Number of blind teats at quarter level	95% CI	Total number of goats/quarters with mastitis (%)	95% CI
Bauchi									
122	244/241	47 (38.5)/60 (24.9)	30-47.8 19.8-30.9	11 (9.0)/14 (5.8)	4.8-15.9 3.3-9.8	3 (1.2)	0.3-3.8	58 (47.5)/74 (30.7)	38.5-56.7 25.0-37.0
Plateau									
65	130/128	19 (29.2)/30 (23.4)	18.9-42 16.6-31.9	5 (7.7)/8 (6.3)	2.9-17.8 2.9-12.3	2 (1.5)	0.2-6.0	24 (36.9)/38 (29.7)	25.6-49.8 22.1-38.5
Edo									
63	126/124	15 (23.8)/26 (21.0)	14.4-36.5/14.4-29.4	4 (6.4)/7 (5.7)	2.1-1.6 2.5-11.7	2 (1.6)	0.3-6.2	19 (30.2)/33 (26.7)	19.6-43.2 19.3-35.4
Total									
250	500/493	81 (32.4)/116 (23.5)	26.7-38.6/19.9-27.6	20 (8.0)/29 (5.9)	5.1-12.3 4.0-8.4	7 (1.4)	0.6-3.0	101 (40.4)/145 (29.4)	34.3-46.8 25.5-33.7

95% CI: 95% Confidence interval

Of the 20 lactating goats with clinical mastitis case, 10 (50%; 95% CI: 2.7-7.2) goats were found to be with serous form of mastitis, 7 (35%; 95% CI: 1.5-5.9) goats with catarrhal, and 3 (15%; 95% CI: 0.4-3.9) goats with purulent-catarrhal. On physical examination, most of the clinical mastitis goats have shown sign of depression (65%), lack of appetite (75%), increase in body temperature (fever) of 40°C and above (100%), PR >90 beats/min (80%), respiratory rate above 25 breaths/min (85%), and only 40% recumbency (Table-2).

**Table-2:** The prevalence of clinical presentations of clinical mastitis with regard to physical examination of the animal at individual level, n=20.

Category	Number of positives (%)	95% CI
Type of clinical mastitis		
Serous	10 (50.0)	2.7-7.2
Catarrhal	7 (35.0)	1.6-5.9
Purulent-catarrhal	3 (15.0)	0.4-3.9
General animal condition		
Depression		
Yes	13 (65.0)	4.1-8.3
No	7 (35.0)	1.6-5.9
Appetite		
Yes	5 (25.0)	0.9-4.9
No	15 (75.0)	5.2-9.0
Recumbency		
Yes	8 (40.0)	2.0-6.3
No	12 (60.0)	3.6-8.0
Temperature rate (°C)		
≤40	0 (0.0)	0.0-2.0
40.1-40.5	8 (40.0)	2.0-6.4
≥40.6	12 (60.0)	3.6-8.0
Pulse rate (/min)		
≤90	4 (20.0)	0.6-4.4
91-100	6 (30.0)	1.3-5.4
≥101	10 (50.0)	2.8-7.2
Respiration rate (/min)		
≤25	3 (15.0)	0.3-3.9
26-30	8 (40.0)	2.0-6.3
≥30	9 (45.0)	2.4-6.8

95% CI: 95% confidence interval

Of the 29 quarters with clinical mastitis case, 15 (51.7%) quarters were found to be serous, 9 (31%) quarters were catarrhal, and 5 (17.2%) quarters were purulent-catarrhal. On physical inspection of the affected glands, 41.4% of the affected quarters were hyperemic, 55.2% and 44.8% of the affected quarters were slightly and grossly enlarged, respectively. On palpation, 82.8% of the affected quarters were warm, 17.2% (hot), 100% (painful), 58.6% (hard) and 100% enlargement of the supramammary lymph nodes were recorded. Abnormalities were noticed in all the milk samples (Table-3).

**Table-3:** The prevalence of clinical presentations of clinical mastitis with regard to physical examination of the animal at quarter level n=29.

Category	Number of positives (%)	95% CI
Type of clinical mastitis		
Serous	15 (51.7)	3.3-7.0
Catarrhal	9 (31.0)	1.6-5.1
Purulent-catarrhal	5 (17.2)	0.6-3.6
Affected quarter sign		
Size		
Normal	0 (0.0)	0.0-1.5
Slightly enlarge	16 (55.2)	3.6-7.3
Grossly enlarge	13 (44.8)	2.7-6.4
Temperature		
Normal	0 (0.0)	0.0-1.5
Cold	0 (0.0)	0.0-1.5
Warm	24 (82.8)	6.4-9.3
Hot	5 (17.2)	0.6-3.6
Painful to palpation		
Yes	29 (100.0)	8.5-10.0
No	0 (0.0)	0.0-1.5
Hardness		
Yes	17 (58.6)	3.9-7.6
No	12 (41.4)	2.4-6.1
Hyperemia		
Yes	12 (41.4)	2.4-6.1
No	17 (58.6)	3.9-7.6
Teat inflammatory swellings		
Yes	20 (69.0)	4.9-8.4
No	9 (31.0)	1.6-5.1
Enlarged supramammary lymph nodes		
Yes	29 (100.0)	8.5-10.0
No	0 (0.0)	0.0-1.5
Abnormality in milk secretion		
Yes	29 (100.0)	8.5-10.0
No	0 (0.0)	0.0-1.5
If yes, how?		
Watery	15 (51.7)	3.3-7.0
Yellowish (curd-like) with tiny flakes and clots	9 (31.0)	1.6-5.1
Thick and foul-smelling	5 (17.2)	0.7-3.6

95% CI: 95% Confidence interval

The bacteriological research results enabled to identify and characterize cultures of microorganisms from milk samples of mastitis goats. Their relative prevalence rate is presented in Table-4.

**Table-4:** The relative prevalence rate of various bacteria isolated from the mastitis milk samples of goats in Nigeria.

Microorganisms	Frequency and percentage of isolates		

	Clinical mastitis, n (%)	Subclinical mastitis, n (%)	Total, n (%)
*Staphylococcus aureus*	15 (7.5)	25 (12.5)	40 (20.0)
*Staphylococcus auricularis*		4 (2.0)	4 (2.0)
*Staphylococcus chromogenes*		9 (4.5)	9 (4.5)
*Staphylococcus caprae*		5 (2.5)	5 (2.5)
*Staphylococcus hyicus*		2 (1.0)	2 (1.0)
*Staphylococcus epidermidis*	4 (2.0)	8 (4.0)	12 (6.0)
*Staphylococcus xylosus*		2 (1.0)	2 (1.0)
*Staphylococcus lentus*		1 (0.5)	1 (0.5)
*Micrococcus luteus*		5 (2.5)	5 (2.5)
*Streptococcus agalactiae*	6 (3.0)	16 (8.0)	22 (11.0)
*Streptococcus dysgalactiae*	1 (0.5)	4 (2.0)	5 (2.5)
*Streptococcus pluranimalium*		5 (2.5)	5 (2.5)
*Streptococcus uberis*	1 (0.5)	3 (1.5)	4 (2.0)
*Streptococcus pneumoniae*		1 (0.5)	1 (0.5)
*Streptococcus ruminatorum*		3 (1.5)	3 (1.5)
*Streptococcus suis*		2 (1.0)	2 (1.0)
*Escherichia coli*	9 (4.5)	22 (11.0)	31 (15.5)
*Enterobacter cloacae*		3 (1.5)	3 (1.5)
*Klebsiella oxytoca*	1 (0.5)	5 (2.5)	6 (3.0)
*Klebsiella pneumoniae*	1 (0.5)	3 (1.5)	4 (2.0)
*Morganella morganii*		4 (2.0)	4 (2.0)
*Salmonella* Typhimurium		4 (2.0)	4 (2.0)
*Proteus vulgaris*	3 (1.5)	7 (3.5)	10 (5.0)
*Citrobacter freundii*		4 (2.0)	4 (2.0)
*Pseudomonas aeruginosa*		2 (1.0)	2 (1.0)
*Acinetobacter rudis*		2 (1.0)	2 (1.0)
*Acinetobacter haemolyticus*		3 (1.5)	3 (1.5)
*Bacillus cereus*	1 (0.5)	4 (2.0)	5 (2.5)
Total	42 (21.0)	158 (79.0)	200 (100.0)

A total of 200 bacterial isolates were recovered from 145 (116 SCM-CMT positive milk and 29 from clinical mastitis quarters) mammary gland secretion samples at quarter level of 104 goats. *S. aureus* (20.0%) was the most frequent isolates from clinical and subclinical mastitic samples. The next most frequent isolates from all bacteria were *E. coli* (15.5%) and *S. agalactiae* (11.0%). Other less common isolates (≤6%) and their frequency of isolation are given in Table-4.

## Discussion

Mastitis, inflammation of mammary gland, is one of the most important diseases of dairy animals worldwide [27-29], and it is notoriously difficult to estimate the losses associated with clinical and SCM, which arises from the costs of treatment, culling, death and decreased milk production, and constituent quality [30]. CM presents significant clinical features of inflammatory signs in udder tissues and abnormal udder secretion, whereas the only indicator of SCM is higher somatic cell count in milk without any visible abnormalities in udder tissue and milk [28]. In dairy goats, the incidence of clinical mastitis may not exceed 5%, while SCM is common and about 6 times more than clinical affections [31].

The present study showed an overall apparent prevalence of mastitis in lactating goats in three Nigerian states to be 40.4% as determined by CMT and clinical examinations of the udder. This finding is relatively higher than most of the previous findings conducted in Nigeria, where the scores range between 10% and 19.1% [30,32,33]; however, it has agreement with the results reported in other countries of the world such as 40.5% in Brazil by Oliveira *et al*. [34] and 43.33% in Bangladesh by Ferdous *et al*. [35]. Besides, the reported apparent prevalence of this study is lower compared to the higher prevalence rate 47.6%, 48.1%, and 61% which were reported in animals in urban areas of Nigeria, Egypt, and Kenya, respectively [14,26,36]. As mastitis is a complex disease involving the interactions of various factors such as differences in management and husbandry practices, geographical distribution, health status of the flock, causative agents, environmental condition (weather), nutritional status and finally the size of the study samples may be the reason for the variation in the prevalence rates between the present study and the previous ones [14,16,30].

In this study, subclinical mastitis has found to be higher than clinical mastitis. Moreover, farmers in Nigeria are not well informed about the silent cases of mastitis. A similar observation of the high dominance of SCM was recorded by several studies [35,37].

Quarter apparent prevalence of mastitis (29.4%) recorded in this study was comparable with the finding of Mdegela *et al*. [38] who reported quarter prevalence rate of 30% but lower than the report made by Pirzada *et al*. [39], Ferdous *et al*. [35], and Moshi *et al*. [40], who reported 38%, 50%, and 72.8 % in Pakistan, Bangladesh, and Tanzania, respectively.

Typically, serous mastitis is acute, and in the absence of its treatment within 10-15 h it may transform to catarrhal form of mastitis, less often to purulent-catarrhal form [21].

The apparent prevalence of blind mammary quarters (1.4%) closely agrees with the result of Bayan *et al*. [41] and Dinaol *et al*. [42] who reported certain case of quarter blindness (unfunctional or not functional quarter) in cow. A lack of screening SCM and late or not treating clinical cases could lead to acquired blindness of mammary gland. Blind mammary quarters contribute to high SCM and loss of milk production with a subsequent impact on food security [43].

The high prevalence of *S. aureus* followed by *E. coli* in this study is in accordance with other workers who found a higher prevalence of these organisms from goat’s milk samples positive for mastitis [11,44]. These findings and other research works justify that *S. aureus* is the most important and predominant mastitis-causing pathogen globally, including Nigeria. The high prevalence of *S*. *aureus* in goats is suggested to be reinfection of mammary glands and teat lesions which transfer during milking operation, and in Nigeria, mostly, hand milking is practiced. Intramammary infections caused by *S*. *aureus* are very important from public health point of view due to its ability to produce thermostable enterotoxins and leukotoxins [45]. The higher prevalence of *E. coli* could be linked to the poor hygienic practices in the dairy environment. This may be attributed to the increased opportunity of infection with time and the prolonged duration of infection, especially in a herd without a mastitis control program [44].

## Conclusion

Mastitis is of great economic importance to milk producers because the disease has a negative impact on several important aspects of goat and herd performance. From the present study, it was concluded that the prevalence occurrence of subclinical form of mastitis is 4 times greater than the clinical form. Moreover, *S*. *aureus*, *E. coli*, and *S. agalactiae* were the most predominant mastitis causative microorganisms.

## Authors’ Contributions

NVP contributed in designing the experiment. FAD conducted the research work. NVP and FAD analyzed the data and drafted the manuscript. Both authors have read and approved the final manuscript.
